# Examinations in Elementary Schools

**Published:** 1889-04-06

**Authors:** 


### The Hospital

A Weekly Institutional Journal of

**Science, Medicine, Nursing, and Philanthropy.**

*For Hospitals, Asylums, Medical Practitioners, Students, Nurses, Families, Parishes, Congregations, and the Charitable Public.*

Vol. VI. No. 132.] [April 6, 1889.

### Examinations in Elementary Schools.

Everything in nature that lives and works is subject to examination, and to acceptation or rejection. The whole world that is known to man is a place of criticism, of judgment, and of decision. He who has a house or a horse to sell judges that it is worth a certain amount; he who wishes to buy the house or the horse also weighs its merits in his critical scales, and decides what value he puts upon it. If a manufacturer produces cloth, or chairs, or bicycles, or surgical instruments, he makes them of a certain standard of excellence. Accordingly, as they reach a high, a medium, or a low standard, they are valued and they are paid for. Examination, judgment of results, is, therefore, an absolutely elementary human proceeding. It is as old as man; it is destined to last as long as man, and no doubt longer.

The examination of children in elementary schools is an important part of State business. It is important because of its extent, and because of the great issues at stake. The number of children examined is counted by millions. The training to which they are subjected, the worth of which it is the object of examinations to ascertain, is the process which is to make of these millions of children good, bad, or middling citizens, industrious and worthy, or idle and worthless men and women. No wise man will therefore undervalue examination. It is the very essence of all right and successful business. If any state or man be destitute of examining power, that state or man belongs to the class of helpless things whose failure is inevitable. These propositions are so self-evident, that it would perhaps be impossible to find any intelligent reader who would think of calling them in question.

But though all men agree upon the absolute necessity of critical examination in all human affairs, opinions may be infinitely diverse as to the methods which ought to be pursued. That is, in fact, exactly what we find in daily experience. School, college, and professional examinations of a thorough and searching character are of comparatively recent date. As the result, partly of this, and partly of the natural difficulties of the subject, there is a fierce conflict constantly raging over every field of examination in which operations have been commenced. Examiners themselves show nothing like unanimity of opinion even upon the leading principles which should guide and define their work. There is perforce some uniformity of actual practice, because when a definite thing has to be done, it must be done somehow and by some method, whether by the best or the worst. But in the present case even the doers of it, or at any rate the more intelligent of them, are aware of the experimental and tentative nature of their operations; and they preserve an open and uncrystallised mind on the subject.

The "New Code of Regulations" which has been recently issued by the Education Department, continues in use for another year a rule which seems to us well worthy of the deepest and most careful reconsideration. The rule is found in Chap. II. under the heading "Inspection," and provides that "the department, at the time of agreeing to place a school on the annual grant list, informs the managers in what month to look for the inspector's annual visit. This month continues the same from year to year, unless the department informs the managers of a change." A rider is appended to the rule, which states that "Notice of the day of the inspector's annual visit is given beforehand to the managers."

That is where we join issue with the department. Our objections are based primarily on the principles of a rational physiology, but they extend into the regions of educational and State economy. To begin with, the department has instituted for young children, from three to thirteen years of age, an annual judgment day; a day for the many of anxiety and dread to be looked forward to, and of disappointment and shame to be looked back upon. The few, no doubt, have their successes and their triumphs. But to the majority of the school the weeks preceding and the weeks following the inspector's visit are weeks of decided worry and unhappiness. The question we have to ask is, Should children of the age in question, and at the immature period of development they have then reached, be subjected to the peculiar kind of mental strain which is inevitably produced by the examination as at present carried out? We say emphatically, "No." Our objection is not, as we have shown, to examination in itself, but to examination at a fixed and known period—to examination, in short, which can be specially "crammed" and prepared for. To the whole system of "cramming" for examinations there are the strongest possible physiological objections, and these we may discuss from time to time as opportunity offers; but what we are concerned with now is the system of "cramming," and of special preparation as it affects Board school children between the ages of three and thirteen or fourteen.

Children under fourteen years of age are, to all intents and purposes, young animals. The body is in a developing condition, the mind and the moral faculty are still embryonic. The objects which should be aimed at with these young creatures are the building up of a strong body, a capable mind, and an honest character. It should be no part whatever of the business of their trainers to burden and harass their minds with mere answers to probable questions. That is injurious, and only injurious if it be carried to such an extent as to produce the slightest degree of mental worry or strain. All the training, both intellectual, moral, and physical, to which children under fourteen are subjected ought to be of a kind that not only puts no strain on their powers, but is well within those powers. To work them at high pressure for days and weeks together is highly dangerous, and ought to be made penal. But when the very month, and week, and day or days of the annual school inspection are fixed from year to year, published at the beginning of the year, and, practically, never altered, it is clear that both the masters and the children can begin weeks, or even months, beforehand to put special strain upon themselves with a view to distinction at the testing time. It is a matter of common knowledge that such strain is put by masters both upon themselves and their pupils for a greater or smaller number of weeks before the inspection day.

Examination and inspection, we have admitted, are imperative; but their sole concern should be to weigh and measure the results of the average work of the year; to find out, not the quantity of knowledge in any given child's mind, but the degree of training, of brightness, and of general capacity and healthiness. In order to ascertain the average quality of the teaching, the period of examination should never be published beforehand, but, on the contrary, should be sedulously concealed. If it be necessary to give the master some notice, that notice should be of the briefest—not more than one or two days—and should on no account give him time to put any special strain on the children. Of course, if this system were carried out, a higher class of school inspectors than are now employed would probably be required. At present a man may become an inspector of schools who merely possesses a qualifying amount of knowledge. Under a better system the higher qualification of intellectual fitness would be demanded. Any University man, however hopelessly stupid, can manage to ascertain whether a boy or girl is able to write and spell and do a few sums in arithmetic, or struggle through a little elementary Latin or French. But to so inspect a school as to discover whether its teacher is a man who illuminates every corner of every child's mind with the bright rays of an honest and discriminating intellect is a different matter. Matthew Arnolds do not grow on every bush, and if they did they are happily not all claimed and devoured by Her Majesty's "Education Department." In the interests of the present generation of children, and of the future every-day work of the State, we commend to those in power and to all concerned these elementary physiological principles and commandments, which are so persistently defied and trampled upon by the present oppressive and altogether injurious system.

